# 416. Evaluating Indwelling Devices and Mortality in Invasive Carbapenem-Resistant Enterobacterales Infections, Atlanta, GA, 2012–2019.

**DOI:** 10.1093/ofid/ofac492.493

**Published:** 2022-12-15

**Authors:** Lucy S Witt, Gillian Smith, Mary Elizabeth Sexton, Monica M Farley, Jesse T Jacob

**Affiliations:** Emory University, Atlanta, Georgia; Georgia Emerging Infections Program, Atlanta, GA; Foundation for Atlanta Veterans Education and Research, Decatur, GA; Atlanta Veterans Affairs Medical Center, Decatur, GA, Atlanta, Georgia; Emory University, Atlanta, Georgia; Emory University, Atlanta, Georgia; Emory University School of Medicine, Atlanta, GA; Georgia Emerging Infections Program, Atlanta, GA, Atlanta, Georgia

## Abstract

**Background:**

Carbapenem-resistant Enterobacterales (CRE) infections pose a grave public health threat due to the potentially silent transmission leading to outbreaks, limited therapeutic options and high mortality. We sought to identify risk factors for mortality in patients with invasive CRE infections and describe the association between indwelling medical devices and 90-day mortality.

**Methods:**

The Georgia Emerging Infections Program performs active population and laboratory based surveillance for CRE in the Atlanta, Georgia metropolitan area. Using this data we created a retrospective observational cohort of patients with invasive CRE infections between 2012 and 2019. Invasive infections were defined as resistant isolates obtained from a normally sterile site (Table 1). Indwelling medical devices, including central venous catheters (CVCs), were present within two calendar days prior to infection.

We completed bivariate analyses examining the relationship between covariates and mortality. Multivariable log binomial regression was used to estimate adjusted risk ratios (aRR) for the association of covariates and all-cause 90-day mortality. Sub-group analyses were completed evaluating only those patients with CVCs and those with at least two indwelling devices.

**Table 1.**
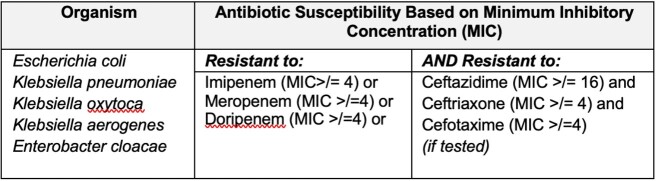
Definition of Carbapenem-resistant Enterobacterales

Abbreviations: MIC – minimum inhibitory concentration

**Results:**

There were 154 invasive CRE infections (Table 2) with 87.7% having at least one indwelling device and an overall mortality of 23.4%. Intensive care unit (ICU) admission, having at least two indwelling devices, and requiring chronic dialysis were associated with mortality on bivariate analysis. The presence of any indwelling device (aRR 1.02, 95% CI 0.36, 2.89) or specifically a CVC (aRR 1.13, 95% CI 0.54, 2.37) were not associated with increased risk of 90-day mortality in unadjusted or multivariable analysis (Table 3). Having at least two indwelling devices was associated with increased risk of mortality (aRR 2.48, 95% CI: 1.02, 5.99) (Table 3).

Characteristics of Patients with Invasive CRE Infections in Atlanta, Georgia 2012-2019

**Figure:**
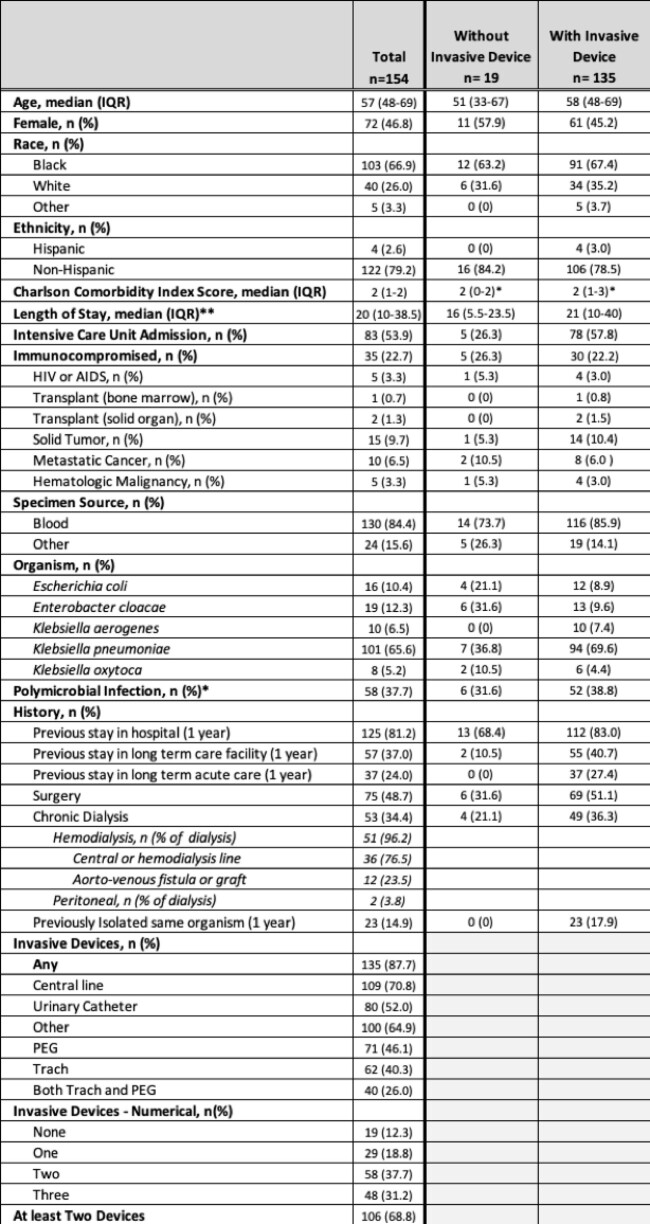

Race: 6 unknowns, Ethnicity: 28 unknowns

Other race: American Indian or Alaska Native, Asian, Native Hawaiian or Pacific Islander

Other sites of infection: deep tissue, sterile fluid, or other sterile site

Other indwelling devices: endotracheal or nasotracheal tube, nephrostomy tube, nasogastric tube, other

* 1 missing

**16 patients never admitted to the hospital and 2 with missing data

Risk Ratios for Invasive Devices and Mortality Including Subgroup Analyses

**Figure:**
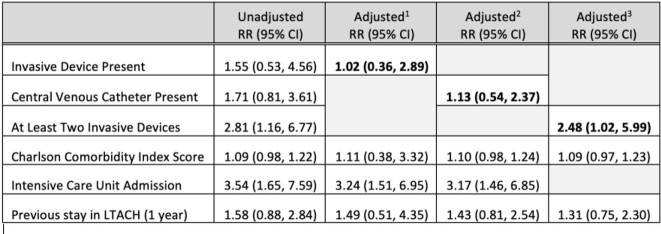

1 Adjusted for indwelling device, Charlson comorbidity score, intensive care admission, previous stay at LTACH

2 Adjusted for central venous catheter, Charlson comorbidity score, intensive care admission, previous stay at LTACH

3 Adjusted for at least two indwelling devices, Charlson comorbidity score, previous stay at LTACH

Abbreviations: LTACH - Long Term Acute Care hospital

**Conclusion:**

In patients with invasive CRE infections, indwelling medical device use was frequent but only associated with mortality in patients with multiple devices. Stewardship of medical devices may be an important target for intervention in this population.

**Disclosures:**

**All Authors**: No reported disclosures.

